# Neoadjuvant outperforms adjuvant regimens in resectable advanced Chinese melanoma patients: lymph node preservation as a key immunological advantage

**DOI:** 10.3389/fimmu.2025.1673308

**Published:** 2025-10-01

**Authors:** Shuguang Hou, Bolun Zhao, Babo Zhang, Luyang Zhao, Hao Zhang, Yang Chen, Wanfu Zhang, Guannan Zhu, Hao Guan

**Affiliations:** ^1^ Department of Burns and Cutaneous Surgery, Xijing Hospital, Fourth Military Medical University, Xi’an, China; ^2^ Department of Dermatology, The 989th Hospital of the Chinese People's Liberation Army Joint Logistic Support Force, Luoyang, China; ^3^ Department of Dermatology, Xijing Hospital, Fourth Military Medical University, Xi’an, China; ^4^ Dermatology Hospital, Southern Medical University, Guangzhou, China

**Keywords:** acral melanoma, lymph node dissection, immunotherapy, adjuvant, neoadjuvant

## Abstract

**Background:**

Regional lymphadenectomy was once considered as the standard treatment for melanoma patients with positive SLNB result, aimed at reducing the risk of recurrence and metastasis. However, recent researches have suggested multiple key roles of regional lymph node clusters in anti-tumor immune responses. Therefore, in the era of immunotherapy, whether lymph node dissection ultimately benefits patients remains to be determined, especially in acral melanoma, the leading subtype in east Asian population, which has a poorer response to immune checkpoint inhibitors (ICIs).

**Methods:**

We retrospectively analyzed 172 patients with resectable advanced melanoma (stage III-IV, M1a) from a tertiary center and categorized them into four groups based on treatment regimens: immunotherapy group, observation group, neoadjuvant group, adjuvant group. In patients receiving immunotherapy (including immunotherapy group, neoadjuvant group, and adjuvant group), anti-PD-1 antibody was given accompanied with interferon-α1b.

**Results:**

With a median follow-up of 18.87 months, multivariable analysis confirmed significantly longer RFS for neoadjuvant versus adjuvant group. The adjusted HR was 2.02 (95% Cl 1.05–3.89, p= 0.035). Numerical improvements over immunotherapy and observation groups did not reach statistical significance, with p-values of 0.120 and 0.073 respectively. The 2-year RFS rate was significantly higher at 39.5% versus 13.8%. Notably, surgery-related adverse events occurred in 50.0% of patients (12.5% grade ≥3), with no significant differences observed among groups.

**Conclusions:**

This study demonstrates that in Chinese patients with advanced melanoma, neoadjuvant therapy is associated with significantly prolonged RFS compared to adjuvant therapy, with suggestive but non-significant advantages against other approaches.

## 1 Introduction

Although immediate completely lymph node dissection (CLND) failed to improve overall survival (OS) in melanoma patients with positive SLNB, it still showed benefits in disease-free survival/melanoma-specific survival (DFS/MSS) for patients with Breslow thickness 1.2–3.5 mm (1, 2). These findings once established CLND as the standard treatment for SLNB-positive patients (3). However, subsequent MSLT-II and DeCOG-SLT trials confirmed that close observation achieved comparable survival outcomes to CLND in SLNB-positive patients (4, 5), while CLND was associated with higher complication rates (6). Based on this evidence, the NCCN guidelines progressively de-escalated their recommendation for CLND, with the 2025 version explicitly listing “active surveillance with regional lymph node ultrasound or imaging alternatives to immediate surgery” as the preferred approach (7).

In the era of immunotherapy, the clinical benefit of CLND for patients with advanced melanoma has been further questioned. Recently, Fransen MF and colleagues proved that resection of tumor-draining lymph nodes significantly impairs antitumor immune responses using *in vivo* model (8). These findings also appear to corroborate some clinical observations: in postoperative recurrent non-small cell lung cancer patients, an elevated dissected lymph node count (cutoff: 16) was associated with poorer immunotherapy efficacy (9). Current evidence suggests that draining lymph nodes serve as a critical foundation for antitumor immunity (10), necessitating a fundamental reevaluation of conventional lymph node dissection—shifting from “questionable clinical benefit” to “potentially detrimental to immunotherapy efficacy. In the PRADO trial, patients who achieved a major pathologic response (MPR) after ipilimumab plus nivolumab therapy maintained a 2-year recurrence-free survival (RFS) of 93% despite omitting therapeutic lymph node dissection (TLND), further supporting the trend toward surgical de-escalation in melanoma management (11).

The Asian population exhibits distinct clinicopathological features: acral melanoma accounts for of cases 50–60% (vs. <5% in Caucasians) (12), which results in earlier lymph node metastasis, inferior survival outcomes (12, 13), and attenuated responses to immunotherapy (14–16). Consequently, the generalizability of cutaneous melanoma-based nodal management to Asian populations warrants further investigation. Here, we assess how different treatment strategies involving lymph node management influence immunotherapy outcomes in Chinese patients with advanced melanoma, addressing this critical knowledge gap to provide evidence for optimized surgical timing in precision management.

## 2 Methods

### 2.1 Study design and patients

This retrospective study included patients with stage III–IV, M1a resectable melanoma (AJCC 8th edition) at Xijing Hospital, Fourth Military Medical University between July 1, 2019 and September 30, 2024. Inclusion criteria: (1) histopathologically confirmed melanoma, staging III–IV, M1a; (2) receive TLND in Department of Burns and Cutaneous Surgery, Xijing Hospital; (3) follow-up duration >6 months; (4) complete medical records available. Exclusion criteria: (1) with a previous history of lymph node dissection; (2) combination management including chemotherapy, radiotherapy, or targeted therapy.

**Figure 1:**
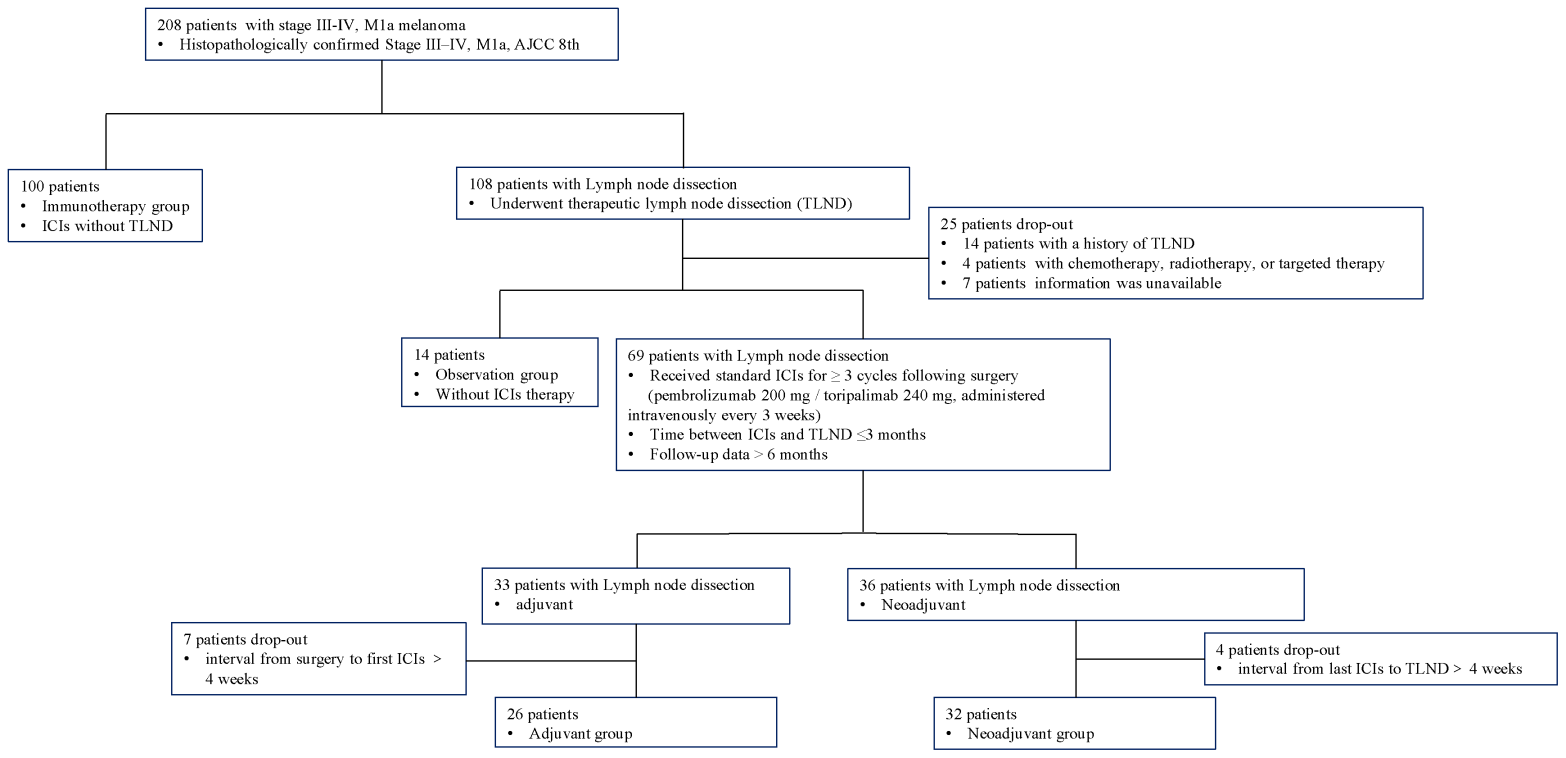
Flowchart of patient selection and grouping.

The eligibility criteria and study flow are detailed in **Figure 1**. This cohort study included four treatment groups: (1)immunotherapy group: patients received immunotherapy only, without TLND; (2)observation group: patients underwent TLND, followed by observation; (3)neoadjuvant group: patient received 3 cycles of immunotherapy before TLND, followed by at least 3 additional postoperative cycles of immunotherapy, the surgery was performed within 4 weeks after the last immunotherapy administration; (4)adjuvant group: patient received at least 3 cycles of immunotherapy after TLND (17).

All patients included in this study were initially diagnosed with technically resectable disease. Resectability was defined as the anatomical feasibility of achieving a complete macroscopic resection (R0) with negative margins, as confirmed by a multidisciplinary team (MDT) based on pretreatment radiological imaging (18). The final treatment is determined through joint decision-making between the physicians and the patients, influenced by factors including clinical practice guideline recommendations, MDT discussion outcomes, the patient’s health insurance coverage, financial capacity, and patients’ personal preferences. In patients receiving immunotherapy (including immunotherapy group, neoadjuvant group, and adjuvant group), ICIs were given accompanied with interferon-α1b (600 ug, subcutaneously administered every other day).

All lymph node dissection procedures were performed by certified and experienced surgeons using standardized techniques, with removal of the involved nodal basins. All excised lymph node specimens were sent for pathological examination and evaluated by at least two board-certified pathologists trained in standardized diagnostic practices. All specimens were independently reviewed by two pathologists. Detailed surgical data are presented in **Supplementary Table 1**.

### 2.2 Efficacy and safety assessment

The primary endpoint was RFS, which was defined as the time from treatment initiation (date of first anti-PD-1 monoclonal antibody infusion for the immunotherapy group or surgical date for the therapeutic lymph node dissection group) to the first documented recurrence (local/lymph nodal/distant) or death from any cause. Data were retrospectively collected through hospital electronic medical records, institutional databases, and structured telephone follow-ups. Per clinical protocols, patients underwent regular clinical/imaging assessments (contrast-enhanced CT/MRI or PET-CT) every 3–6 months post-treatment, with extended intervals permitted for long-term recurrence-free cases; all data were systematically recorded. Recurrence required meeting ≥1 criterion (prioritizing histologic confirmation when feasible): (1) radiographic progression per RECIST 1.1 (new lesions or ≥20% increase in target lesions), (2) biopsy-confirmed metastasis (unless contraindicated), or (3) multidisciplinary consensus (≥1 medical oncologist, radiologist, and pathologist) for equivocal cases.

Adverse events were graded according to the Common Terminology Criteria for Adverse Events (CTCAE v5.0). This study was conducted in compliance with the Declaration of Helsinki, approved by the Ethics Committee of the Fourth Military Medical University (Approval No: KY20242252-F-1).

### 2.3 Statistical analyses

Continuous variables were summarized as medians (interquartile ranges), and categorical variables as frequencies (percentages). Intergroup differences were assessed using Pearson’s χ² test (or Fisher’s exact test for expected cell counts <5) for categorical variables and the Mann-Whitney U test for continuous variables, as appropriate. RFS was estimated via the Kaplan-Meier method, with between-group comparisons performed using the log-rank test. Multivariable Cox proportional hazards regression was employed to adjust for prespecified confounders. All statistical tests were two-sided, with p < 0.05 considered significant. Analyses were conducted using IBM SPSS Statistics (Version 26.0) and R 4.4.3.

A multivariable Cox proportional hazards model was used to compare RFS between groups. The model adjusted for the following pre-specified covariates: age, sex, primary site, stage, Breslow thickness, ulceration, gene mutation, and ICIs type. The proportional hazards assumption was verified using Schoenfeld residuals. The global test yielded a p-value of 0.098. Specifically, the treatment variable yielded a p-value of 0.370, indicating no significant violation of the proportional hazards assumption for any covariate in the model (see **Supplementary Figure 1**).

## 3 Results

### 3.1 Baseline characteristics

This study enrolled a total of 172 patients with stage III to IV, M1a melanoma, who were categorized into four groups according to different treatments. One hundred patients received immunotherapy without surgery, 32 underwent neoadjuvant therapy while 26 received adjuvant therapy, 14 patients received close observation after surgery. Baseline characteristics including age, primary site, clinical stage, Breslow thickness, ulceration status, and driver gene mutations were comparable across all groups (all P>0.05). Significant differences were observed in sex distribution (P<0.01) and types of ICIs(P<0.01) (**Table 1**).

**Table 1:** Baseline profiles.

Variable	Immunotherapy N=100	Adjuvant N=26	Neoadjuvant N=32	Observation N=14	P value
Age (median [IQR])	60 [48–69]	57 [38–63]	56[46-69]	60[46-69]	
Age					
≤60	58 (58%)	16 (61.5%)	19 (59.4%)	6 (42.9%)	0.692
>60	42 (42%)	10 (38.5%)	13 (40.6%)	8 (57.1%)	
Sex					
Male	59 (59%)	6 (23.1%)	19 (59.4%)	8 (57.1%)	0.01
Female	41 (41%)	20 (76.9%)	13 (40.6%)	6 (42.9%)	
Stage					
III a-b	11 (11%)	4 (15.4%)	6 (18.8%)	0 (0%)	0.335
III c-d	77 (77%)	20 (76.9%)	24 (75.0%)	14 (100%)	
IV M1a	12 (12%)	2 (7.7%)	2 (6.2%)	0 (0%)	
Primary site					
cutaneous	25 (25%)	12(46.2%)	8(25.0%)	3 (21.4%)	0.104
acral	73 (73%)	14 (53.8%)	23 (71.9%)	9 (64.3%)	
other	2 (2%)	0	1 (3.1%)	2 (14.3%)	
Gene mutation					
wild type	37 (37%)	9 (34.6%)	18 (56.3%)	8 (57.1%)	0.220
*BRAF*	21 (20%)	9 (34.6%)	9 (28.1%)	2 (14.3%)	
*NRAS*	23 (23%)	5 (19.2%)	2 (6.3%)	1 (7.1%)	
*cKIT*	10 (11%)	1 (3.8%)	1 (3.1%)	0 (0%)	
unknown	9 (9%)	2 (7.7%)	2 (6.3%)	3 (21.4%)	
Breslow Thickness					
≤4mm	51 (51%)	12 (46.2%)	12 (37.5%)	2 (14.3%)	0.102
>4mm	44 (44%)	12 (46.2%)	19 (59.4%)	10 (71.4%)	
unknown	5 (5%)	2 (7.7%)	1 (3.1%)	2 (14.3%)	
Ulceration					
No	15 (15%)	5 (19.2%)	5 (15.6%)	3 (21.4%)	0.798
Yes	80 (80%)	18 (69.2%)	25 (78.1%)	10 (71.4%)	
unknown	5 (5%)	3 (11.5%)	2 (6.3%)	1 (7.1%)	
ICIs type					
Pembrolizumab	21 (21%)	13 (50%)	11 (34.4%)	/	0.01
Toripalimab	79 (79%)	13 (50%)	21 (65.6%)	/	

### 3.2 Efficacy

#### 3.2.1 RFS outcomes

##### 3.2.1.1 Impact of lymph node dissection on immunotherapy efficacy

**Figure 2:**
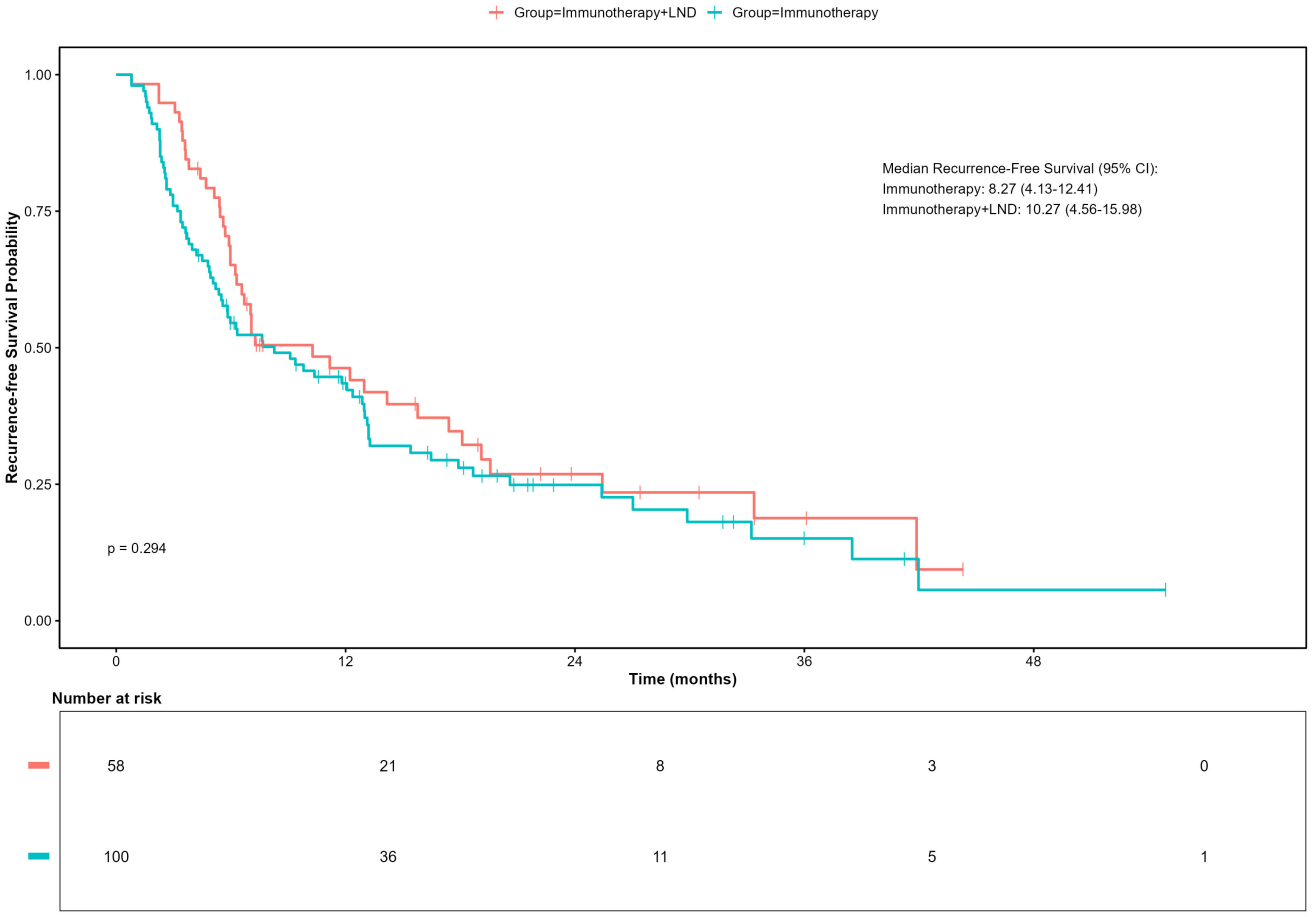
Recurrence-free survival (RFS) in patients receiving immunotherapy with or without lymph node dissection (LND).

As of April 30, 2025, the median follow-up time for the entire cohort was 18.87 months (IQR: 11.83-31.21). Firstly, we compared the RFS of patients who received immunotherapy alone (n=100) with those who received a combination of immunotherapy and lymph node dissection (neoadjuvant and adjuvant group, n=58). In a univariate, unadjusted analysis, the Kaplan-Meier curve and log-rank test demonstrated no significant difference in RFS between these two strategies (p = 0.298, Log-rank). The median RFS was 8.27 months (95% CI, 4.13–12.41) in the immunotherapy group versus 12.97 months (95% CI, 6.53–19.41) in the combination therapy group (**Figure 2**).

##### 3.2.1.2 Impact of surgical timing in patients undergoing dissection

We next focused on the cohort of patients who underwent lymph node dissection to evaluate the impact of surgical timing on the treatment efficacy.

**Figure 3:**
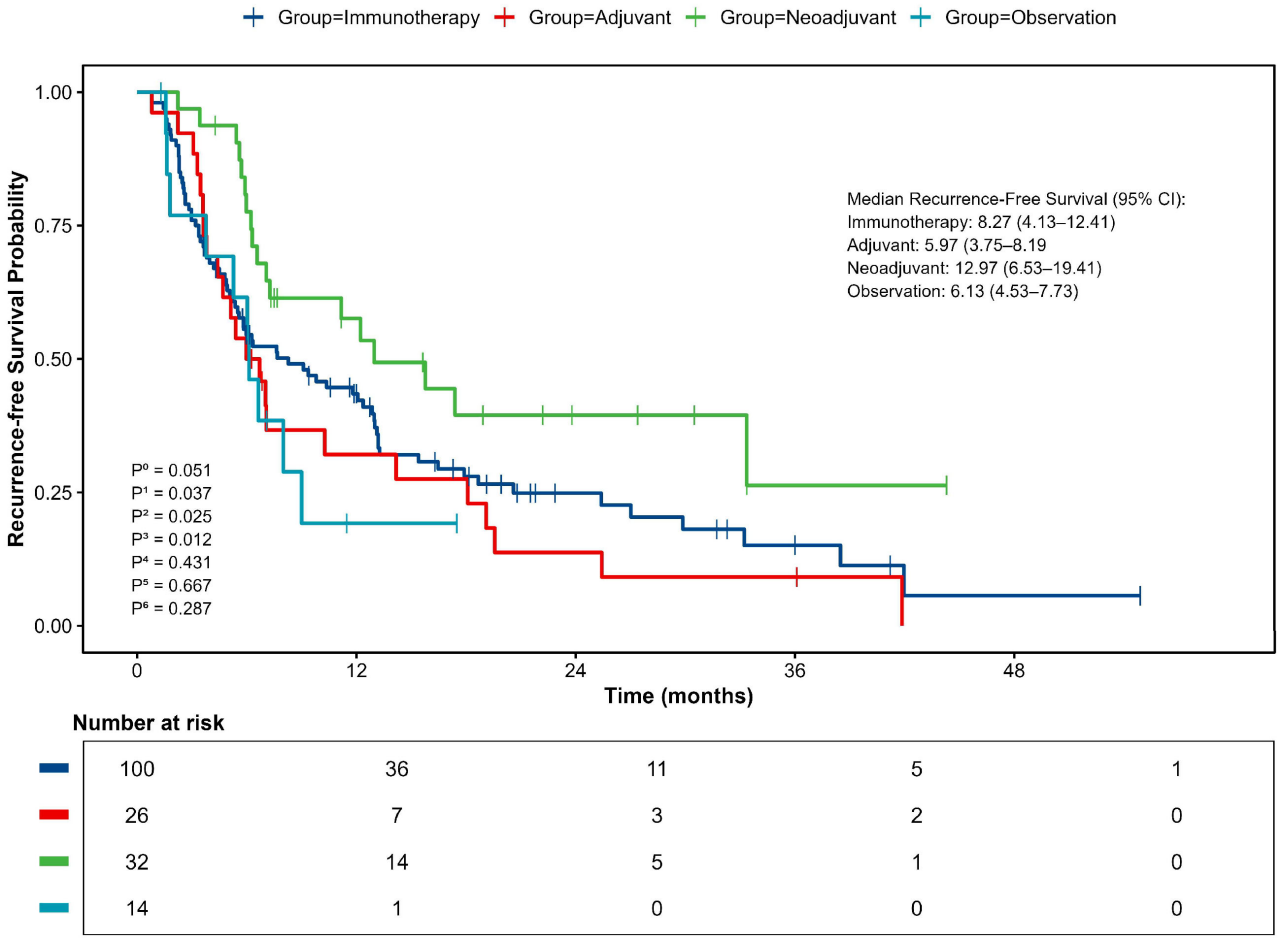
Kaplan–Meier curve for Recurrence-free survival (RFS). P^0^, comparison between 4 groups; P^1^, neoadjuvant vs immunotherapy; P^2^, neoadjuvant vs observation; P^3^, neoadjuvant vs adjuvant; P^4^, adjuvant vs immunotherapy; P^5^, adjuvant vs observation; P^6^, observation vs immunotherapy.

Kaplan-Meier analysis indicated a clear difference in RFS among the treatment groups, though it did not reach statistical significance (p=0.051, **Figure 3**). In unadjusted analyses, the neoadjuvant-adjuvant group showed the most favorable clinical outcomes, with a median RFS of 12.97 months (95% CI 6.53–19.41). This was significantly longer than that observed in the adjuvant group (5.97 months; 95% CI 3.75–8.19; p=0.012). Numerical trends suggesting improved RFS were also observed when compared to the immunotherapy group (8.27 months; 95% CI 4.13–12.41; p=0.037) and the observation group (6.13 months; 95% CI 4.53–7.73; p=0.025). No other pairwise comparisons showed statistically significant differences.

Consistent with the median RFS, analysis of survival rates at specific timepoints showed that the neoadjuvant-adjuvant group had a 1-year RFS rate of 57.6%. This was significantly higher than the observation group (19.2%, p = 0.009, z-test) and showed a strong trend toward significance compared to the adjuvant group (32.1%, p=0.053, z-test). The 2-year RFS rate was 39.5% for the neoadjuvant group, which was significantly higher than that of the adjuvant group (13.8%, p=0.035, z-test). All patients in the observation group experienced recurrence within 2 years.

#### 3.2.2 Multivariable Cox regression analysis

**Figure 4:**
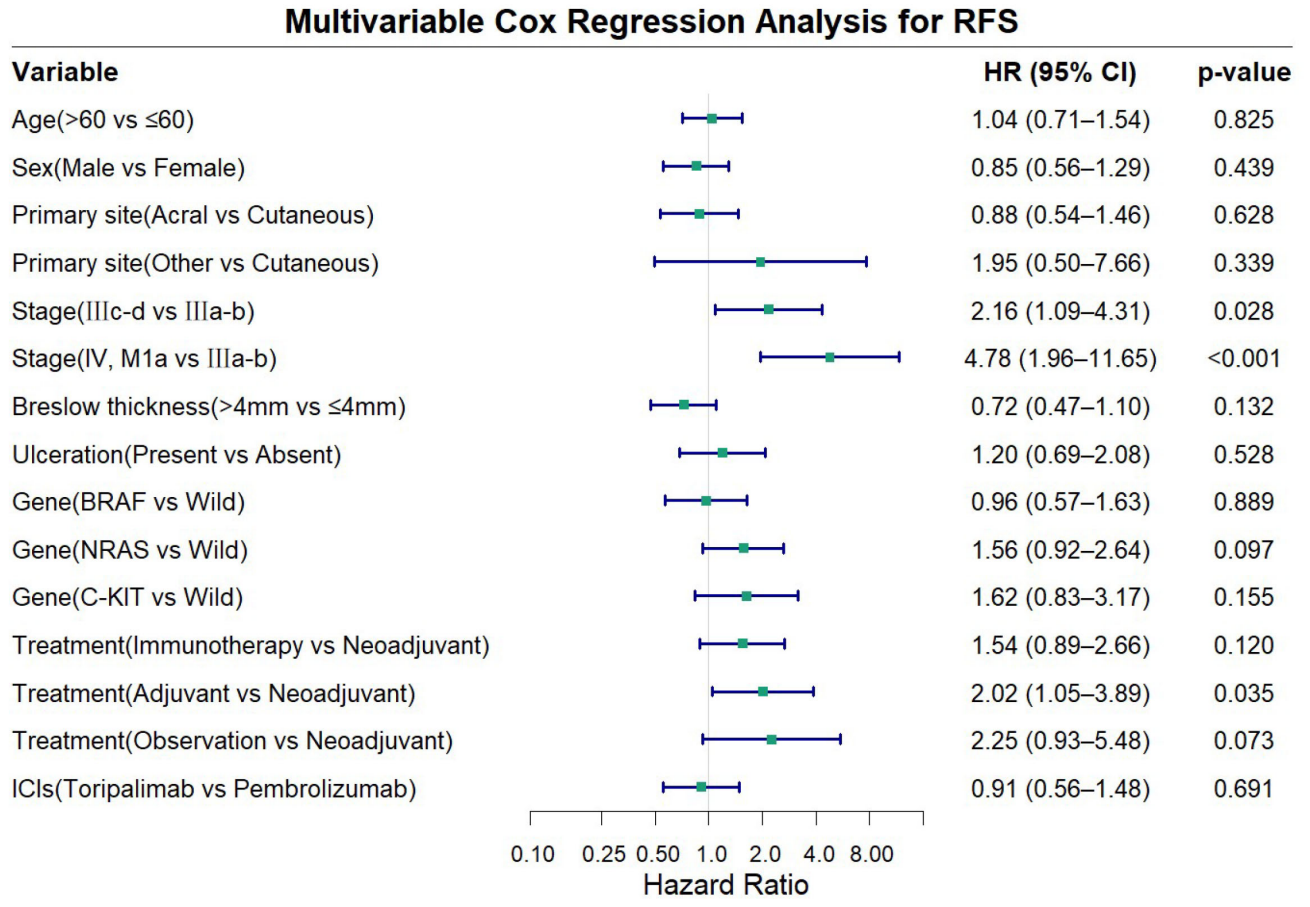
Forest plot showing multivariable cox regression analysis for recurrence-free survival (RFS).

Multivariable Cox proportional hazards analysis adjusting for age, sex, primary site, stage, Breslow thickness, ulceration, gene alterations, ICIs type, and treatment demonstrated that advanced disease stage and treatment modality were independent risk factors for RFS in advanced melanoma (**Figure 4**). Compared to stage IIIa-b disease, stage IIIc-d was associated with a significantly worse RFS (HR = 2.16, 95% CI: 1.09-4.31, P = 0.028), while stage IV, M1a patients exhibited an even higher risk (HR = 4.78, 95% CI: 1.96-11.65, P<0.001). Compared with the other three treatment approaches, the neoadjuvant group showed longer RFS, although statistical significance was achieved only in the comparison with the adjuvant group (HR = 2.02, 95% CI: 1.05-3.89, P = 0.035). Other examined factors did not show statistically significant association with RFS in the multivariate model (all P>0.050).

#### 3.2.3 Pathological response in the neoadjuvant therapy group

Among the 32 melanoma patients who received neoadjuvant therapy followed by lymph node dissection, the pathological responses were as follows: Pathological Complete Response (pCR): 6 cases (18.8%). Pathological Partial Response (pPR): 4 cases (12.5%). Pathological No Response (PNR): 22 cases (68.8%). Based on these data, the Pathological Response (PR) rate—defined as the proportion of patients with ≤50% viable tumor cells (pCR + pPR)—was 31.3% (10/32). Interestingly, the pathologically confirmed positive lymph node rate in patients who did not receive immunotherapy was as high as 90% (36/40). Detailed lymph node dissection-related data, including the anatomical extent of dissection, number of lymph nodes harvested, and the ratio of positive lymph nodes, are provided in **Supplementary Table 2**.

### 3.3 Safety

The incidence of immune-related adverse events (irAEs) was evaluated in the immunotherapy, neoadjuvant, and adjuvant groups while the surgery-related adverse events were analyzed in patients underwent TLND. The results demonstrated no statistically significant differences in the occurrence of either any-grade irAEs or grade ≥3 irAEs between treatment groups (Fisher’s exact test, **Table 2**). Among the 72 patients who underwent TLND, the overall incidence of surgery-related adverse events was 50.0%, with grade ≥3 surgery-related adverse events occurring in 12.5% of cases (as graded by CTCAE v5.0 criteria). No significant differences in surgery-related adverse events rates were observed among the different surgical groups (observation vs adjuvant vs neoadjuvant; all p>0.05). Lymphoedema following TLND emerged as the most frequent surgical complication (incidence 26.4%), with detailed characteristics of other complications presented in **Table 3**.

**Table 2:** Immunotherapy-related adverse events.

Adverse Event	Immunotherapy (N = 100)		Adjuvant (N = 26)		Neoadjuvant (N = 32)		P value	
	Any grade	≥Grade 3	Any grade	≥Grade 3	Any grade	≥Grade 3	Any grade	≥ Grade 3
Any (irAEs)	99 (99%)	26 (26%)	25 (96.2%)	8 (30.8%)	32 (100%)	11 (34.4%)	0.343	0.633
Fever	56 (56%)	0 (0%)	10 (38.5%)	0 (0%)	13 (40.6%)	0 (0%)		
Rash	42 (42%)	4 (4%)	8 (30.8%)	2 (7.7%)	15 (46.9%)	6 (18.8%)		
Oral ulcer	33 (33%)	2 (2%)	3 (11.5%)	1 (3.8%)	6 (18.8%)	0 (0%)		
ALT/AST increased	33 (33%)	3 (3%)	11 (42.3%)	0 (0%)	13 (40.6%)	3 (9.4%)		
Decreased appetite	30 (30%)	5 (5%)	6 (23.1%)	2 (7.7%)	5 (15.6%)	1 (3.1%)		
Fatigue	31 (31%)	5 (5%)	4 (15.4%)	0 (0%)	3 (9.4%)	0 (0%)		
Vitiligo	27 (27%)	0 (0%)	8 (30.8%)	0 (0%)	9 (28.1%)	0 (0%)		
Thyroid dysfunction	26 (26%)	0 (0%)	12 (46.2%)	0 (0%)	6 (18.8%)	0 (0%)		
WBC count decrease	22 (22%)	1 (1%)	9 (34.6%)	0 (0%)	10 (31.3%)	1 (3.1%)		
PLT decrease	14 (14%)	1 (1.1%)	2 (7.7%)	0 (0%)	5 (15.7%)	0 (0%)		
Vomiting	11 (11%)	2 (2%)	1 (3.8%)	1 (3.8%)	1 (3.1%)	0 (0%)		
Arthralgia	11 (11%)	3 (3%)	3 (11.5%)	1 (3.8%)	1 (3.1%)	0 (0%)		
Lose hair	8 (8%)	0 (0%)	2 (7.7%)	0 (0%)	0 (0%)	0 (0%)		
Diarrhea	8 (8%)	0 (0%)	2 (7.7%)	0 (0%)	3 (9.4%)	0 (0%)		
Weight loss	6 (6%)	0 (0%)	0 (0%)	0 (0%)	0 (0%)	0 (0%)		
Nausea	5 (5%)	0 (0%)	1 (3.8%)	1 (3.8%)	1 (3.1%)	0 (0%)		
Pruritus	5 (5%)	0 (0%)	2 (7.7%)	0 (0%)	5 (15.7%)	0 (0%)		
Glucose increase	5 (5%)	0 (0%)	0 (0%)	0 (0%)	1 (3.1%)	0 (0%)		
Autoimmune pneumonia	4 (4%)	0 (0%)	1 (3.8%)	0 (0%)	0 (0%)	0 (0%)		
Hypertension	3 (3%)	0 (0%)	0 (0%)	0 (0%)	1 (3.1%)	0 (0%)		
Headache	2 (2%)	0 (0%)	0 (0%)	0 (0%)	1 (3.1%)	0 (0%)		
Hyperuricemia	1 (1%)	0 (0%)	1 (3.8%)	0 (0%)	0 (0%)	0 (0%)		
Renal dysfunction	1 (1%)	0 (0%)	1 (3.8%)	0 (0%)	1 (3.1%)	0 (0%)		

**Table 3:** Surgery-related adverse events.

Adverse Event	Total (n=72)		Adjuvant (n=26)		Neoadjuvant (n=32)		Observation (n=14)		P value	
	Any grade	≥Grade 3	Any grade	≥Grade 3	Any grade	≥Grade 3	Any grade	≥Grade 3	Any grade	≥ Grade 3
Any	36 (50.0%)	9 (12.5%)	16 (61.5%)	4 (15.4%)	14 (43.8%)	4 (12.5%)	6 (42.9%)	1 (7.1%)	0.663	0.889
Lymphedema	19 (26.4%)	3 (4.2%)	10 (38.5%)	1 (3.8%)	7 (21.9%)	0 (0%)	2 (14.3%)	0 (0%)		
Hematoma /Seroma	9 (12.5%)	2 (2.8%)	3 (11.5%)	1 (3.8%)	4 (12.5%)	1 (3.1%)	2 (14.3%)	0 (0%)		
Lymphatic Fistula	8 (11.1%)	2 (2.8%)	3 (11.5%)	1 (3.8%)	4 (12.5%)	1 (3.1%)	1 (7.1%)	0 (0%)		
Surgical Site Infection	5 (6.9%)	2 (2.8%)	0 (0%)	0 (0%)	3 (9.4%)	1 (3.1%)	2 (14.3%)	1 (7.1%)		
Paresthesia	2 (2.8%)	0 (0%)	1 (3.8%)	0 (0%)	1 (3.1%)	0 (0%)	0 (0%)	0 (0%)		
Venous Thrombosis	1 (1.4%)	1 (1.4%)	1 (3.8%)	1 (3.8%)	0 (0%)	0 (0%)	0 (0%)	0 (0%)		
Flap necrosis	1 (1.4%)	1 (1.4%)	0 (0%)	0 (0%)	1 (3.1%)	1 (3.1%)	0 (0%)	0 (0%)		

## 4 Discussion

Completion lymph node dissection has been shown to bring little improvement to advanced melanoma patients in long-term outcomes (4, 5), and may even compromise the efficacy of immunotherapy (19). Therefore, de-escalation of lymph node dissection has become a reasonable therapeutic approach (7, 20). However, while TLND is undergoing de-escalation globally (21), the unique characteristics of Chinese melanoma patients—including their higher nodal metastasis rates (12, 13, 19) and distinct immunotherapy responses (14–16, 21)—suggest retained therapeutic value of lymph node dissection in this population., compel us to re-evaluate this approach in our population. Therefore, further investigation into the impact of lymph node dissection in the era of immunotherapy is critically important for developing individualized and precision-based treatment strategies for Chinese melanoma patients.

The key finding is that while adding TLND to immunotherapy did not significantly improve RFS, the timing of surgery relative to immunotherapy proved critical. Biologically, although resection may reduce tumor burden, extensive lymph node dissection risks removing crucial sites for immune cell activation—impairing systemic antitumor responses. Our results support this latter mechanism, suggesting that TLND’s theoretical benefits did not translate into survival gains, possibly due to this immunological compromise or confounding prognostic factors. The observed pathological response rate after neoadjuvant therapy further supports the role of immunotherapy underlying the clinical benefit of sequenced treatment.

The PRADO trial provides compelling evidence for de-escalating therapeutic lymph node dissection (TLND) in patients who achieve a major pathologic response to immunotherapy. However, the generalizability of this strategy to our patient population remains uncertain. In our cohort, a substantial proportion of patients did not achieve a sufficient pathologic response. This finding underscores the distinct tumor biology and differential treatment outcomes in this demographic. Consequently, TLND may retain clinical utility for regional disease control in Chinese patients with melanoma, necessitating further investigation within the context of this specific subtype.

This study is the first to demonstrate in Chinese melanoma patients that the clinical outcomes of the neoadjuvant group were significantly better than those of the adjuvant group, align with the SWOG 1801 findings that who received pembrolizumab both before and after surgery demonstrated superior event-free survival (EFS) over adjuvant pembrolizumab alone - although acral melanomas comprised <3% (9/313) of cases in that trial. Importantly, our study confirmed that this therapeutic advantage persists even in Chinese melanoma patients with traditionally lower response rates to immune checkpoint inhibitors (22).

We speculate that this discrepancy may stem from the high proportion of acral melanoma in the Chinese population, its aggressive nature, and early metastatic tendency. Interestingly, the pathologically confirmed positive lymph node rate in patients who did not receive immunotherapy was as high as 90% (36/40), significantly exceeding previously reported rates (23–25). On one hand, this reflects the early metastatic characteristics of acral melanoma; on the other hand, it is closely related to the patient population in this study—over 80% were stage IIIc or later, indicating that patients underwent “therapeutic lymph node dissection”, which aligns more closely with real-world clinical practice.

This phenomenon may have an immunological basis: the antitumor effect of ICIs relies on T-cell activation within tumor-draining lymph nodes (8). As the central hub of antitumor immune responses (26), intact draining lymph nodes facilitate more effective expansion of tumor-reactive T-cell clones (27). Conversely, performing TLND before immunotherapy not only removes this critical immune response site but may also impair the efficacy of subsequent ICIs due to excessive lymph node clearance (9). For Chinese melanoma patients, the neoadjuvant immunotherapy strategy may represent the current optimal choice. Immunotherapy provides powerful systemic control, while subsequent surgery may add value in the following ways: addressing potentially immunotherapy-resistant clones in the primary lesion and regional lymph nodes. Providing critical rescue treatment for patients with a poor pathological response to neoadjuvant therapy. Therefore, the “immunotherapy-first, TLND-second” sequence not only has a theoretical immunological basis but is also supported by clinical data in this Chinese cohort, providing important evidence for optimizing the timing of TLND in melanoma patients.

This conclusion appears to differ from some reported literature (28, 29), which suggests that TLND does not affect the efficacy of adjuvant therapy. We hypothesize that this discrepancy may arise from the failure to distinguish immunotherapy from other adjuvant treatments (e.g., targeted therapy), which is why our study excluded patients who received targeted therapy, radiation, or other non-immunotherapy treatments before recurrence to ensure study homogeneity.

Regarding safety, TLND did not significantly increase the risk of irAEs. The incidence of surgery-related adverse events was 50.0%, with lymphedema (26.4%) being the most common complication. However, as this was a retrospective study, some mild adverse events (e.g., asymptomatic effusions or those not requiring intervention) may have been incompletely recorded, potentially leading to an underestimation of surgery-related adverse events rates.

This study has several limitations that should be considered when interpreting the results. As a single-center retrospective study conducted in Northwest China, our cohort may not be fully representative of the broader melanoma population, thereby limiting the generalizability of our findings. The sample size, particularly in subgroup analyses, was relatively small, reducing statistical power and increasing the risk of Type II error. Significant selection bias may also have been introduced through non-random treatment allocation, influenced by regional healthcare policies, socioeconomic factors, and patient preferences. A substantial proportion of patients chose immunotherapy alone due to concerns about surgical complications, preferences for minimally invasive treatment, financial constraints, or insurance coverage limitations—reflecting underlying disparities in access to multidisciplinary care.

The median follow-up period of 18.9 months was insufficient to evaluate overall survival (OS), so recurrence-free survival (RFS) was used as the primary endpoint. The retrospective design also prevented systematic collection of serial biomarker data, such as paired tissue or blood samples across treatment timepoints, limiting assessment of dynamic changes in the tumor immune microenvironment or identification of predictive biomarkers. The higher rate of pathologically positive lymph nodes observed in our cohort compared to other studies requires further validation in independent datasets. These limitations highlight the need for future multi-institutional prospective studies with broader geographic representation and integrated translational components to validate the impact of lymph node dissection timing and better control for socioeconomic, clinical, and immunologic variables. Such studies should include standardized biomarker collection protocols and extended follow-up to facilitate robust evaluation of survival and correlative biological endpoints.

In summary, our findings suggest that performing TLND after ICI therapy is associated with improved RFS in Chinese melanoma patients, indicating that preservation of tumor-draining lymph node architecture prior to immunotherapy may enhance treatment efficacy. In contrast, upfront TLND before ICI initiation appears to be associated with poorer outcomes. These results are supported by clinical observations and immunological mechanisms. The relatively high incidence of surgery-related adverse events further underscores the need for caution when considering TLND prior to immunotherapy. Additionally, differential treatment responses may be influenced not only by TLND timing but also by distinct biological features of acral melanoma, which is predominant in the Chinese population.

Collectively, these results emphasize the critical role of immunotherapy and lymph node dissection sequencing in treatment outcomes, particularly in populations with unique melanoma subtypes and disease biology. Further validation through larger, multicenter prospective studies is essential to better inform individualized treatment strategies for melanoma patients in China and other Asian populations.

## Data Availability

The raw data supporting the conclusions of this article will be made available by the authors, without undue reservation.
