# Supplementary material for: Neoadjuvant outperforms adjuvant regimens in resectable advanced Chinese melanoma patients: lymph node preservation as a key immunological advantage
Source: Front Immunol. 2025 Oct 1;16:1673308. doi: 10.3389/fimmu.2025.1673308 (PMC12521139; doi:10.3389/fimmu.2025.1673308)
Supplement: Supplementary file 1 [file Table1.docx]

**Supplementary Table 1** Data on Lymph Node Dissection

|  | **Adjuvant**  **n=26** | **Neoadjuvant**  **n=32** | **Observation**  **n=14** | **P-value** |
| --- | --- | --- | --- | --- |
| **Lymph node dissection site** |  |  |  |  |
| inguina | 16 (61.5%) | 24 (75%) | 11 (78.6%) | 0.479 |
| axilllary | 8 (30.8%) | 7 (21.9%) | 2 (14.3%) |  |
| Other | 2 (7.7%) | 1 (3.1%) | 1 (7.1%) |  |
| **Number of dissected LNs** |  |  |  |  |
| ＜10 | 4 (15.4%) | 6 (18.8%) | 3(21.4%) | 0.452 |
| 10-20 | 17 (65.4%) | 24 (75.0%) | 10(71.4%) |  |
| ＞20 | 5 (19.2%) | 2 (6.3%) | 1 (7.1%) |  |
| **Positive number of dissected LNs** |  |  |  |  |
| 0 | 2 (7.7%) | 6 (18.8%) | 2 (14.3%) | 0.008 |
| 1 | 8 (30.8%) | 18 (56.3%) | 7 (50.0%) |  |
| 2-3 | 4 (15.4%) | 5 (15.6%) | 3 (21.4%) |  |
| ≥4 | 12 (46.2%) | 3 (9.4%) | 2 (14.3%) |  |

**Supplementary Table 2** Detailed Pathological Response Rates in the Neoadjuvant Cohort

|  | **ID** | **stage** | **Pathological response** |  | **ID** | **stage** | **Pathological response** |
| --- | --- | --- | --- | --- | --- | --- | --- |
| 1 | PT-001 | ⅢC  ⅢC  ⅢC  ⅢB  ⅢB  ⅢC  ⅢC  ⅢC  ⅢC  ⅢB  ⅢD  ⅢC  Iva  ⅢD  ⅢC  ⅢC | pCR | 17 | PT-047 | ⅢC | pNR |
| 2 | PT-010 | ⅢC | pNR | 18 | PT-050 | ⅢC | pNR |
| 3 | PT-013 | ⅢC | pNR | 19 | PT-055 | ⅢD | pCR |
| 4 | PT-014 | ⅢB | pNR | 20 | PT-057 | ⅢC | pNR |
| 5 | PT-016 | ⅢB | pCR | 21 | PT-060 | ⅢC | pPR |
| 6 | PT-020 | ⅢC | pNR | 22 | PT-063 | Ⅳ, M1a | pCR |
| 7 | PT-022 | ⅢC | pNR | 23 | PT-068 | ⅢC | pNR |
| 8 | PT-024 | ⅢC  60  63  68  69  70  71  72  75  77  78  85  89 | pNR | 24 | PT-069 | ⅢC | pNR |
| 9 | PT-029 | ⅢC | pPR | 25 | PT-070 | ⅢB | pNR |
| 10 | PT-032 | ⅢB | pNR | 26 | PT-071 | ⅢC | pNR |
| 11 | PT-034 | ⅢD | pNR | 27 | PT-072 | ⅢD | pNR |
| 12 | PT-035 | ⅢC | pNR | 28 | PT-075 | ⅢC | pPR |
| 13 | PT-036 | Ⅳ, M1a | pNR | 29 | PT-077 | ⅢB | pNR |
| 14 | PT-039 | ⅢD | pNR | 30 | PT-078 | ⅢB | pCR |
| 15 | PT-045 | ⅢC | pPR | 31 | PT-085 | ⅢC | pNR |
| 16 | PT-046 | ⅢC | pNR | 32 | PT-089 | ⅢC | pCR |


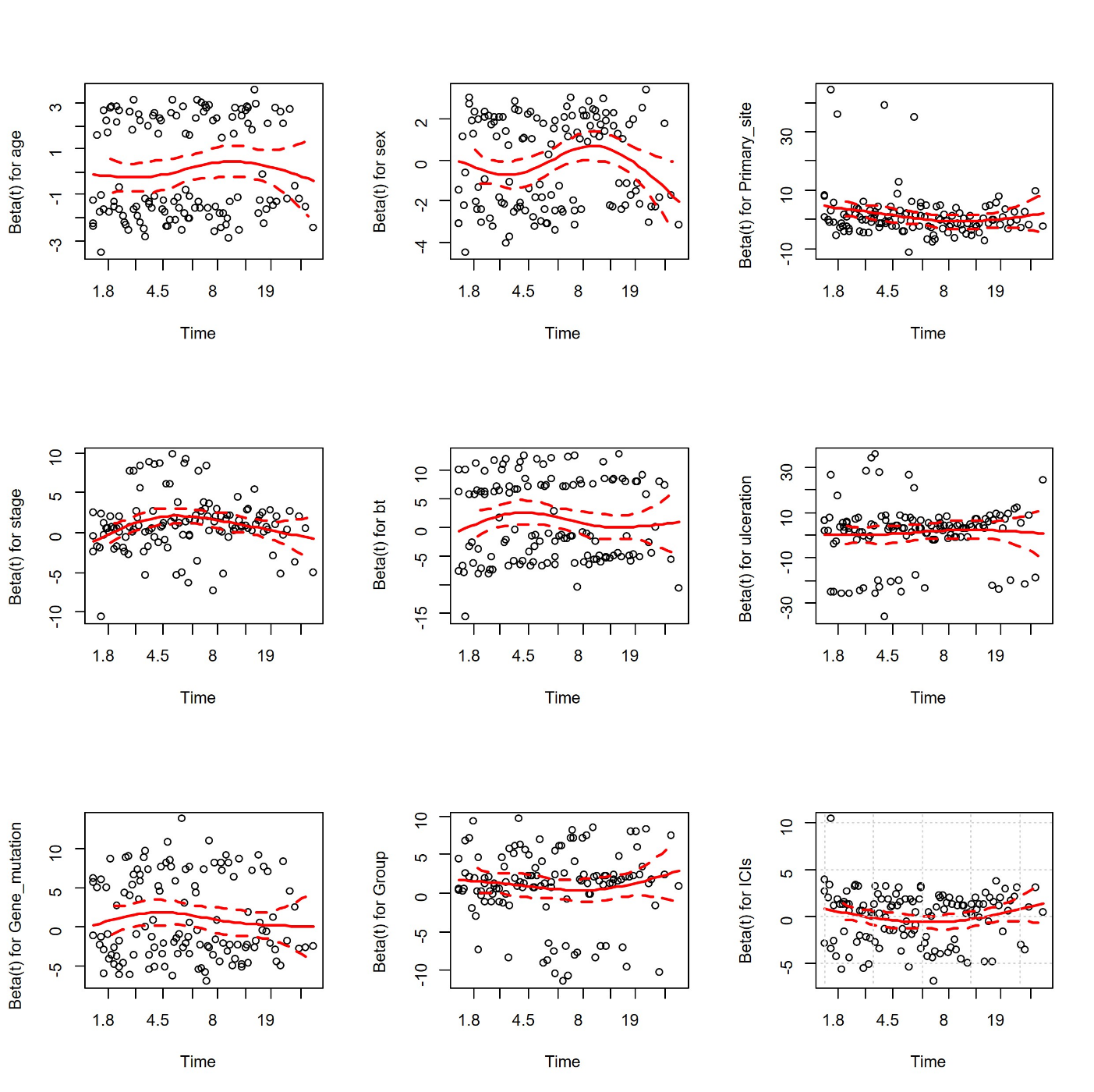


**Supplementary Figure 1:** Assessment of proportional hazards assumptions using time-varying coefficients for recurrence-free survival.

The plots show the estimated time-varying coefficients β(t) for age, sex

, primary site, clinical stage, breslow thickness, ulceration, gene mutations, ICIs type, and group(treatment) from a flexible Cox regression model. A stable, horizontal trend would indicate that the proportional hazards assumption holds, while a non-flat trend suggests the hazard ratio changes over time. The solid lines represent the point estimates, shaded areas denote 95% confidence intervals, and the dashed line at β(t) = 0 represents no effect.


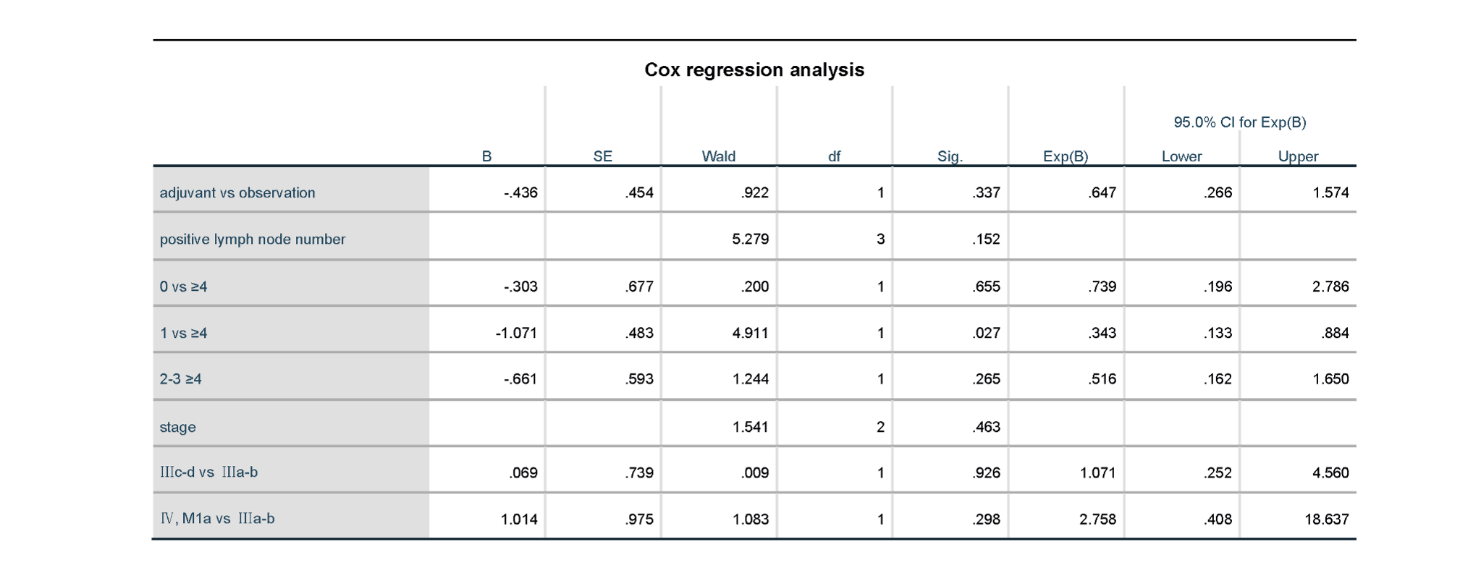


**Supplementary Figure 2** Multivariable analysis of recurrence-free survival. Cox regression analysis in the surgery subgroup (adjuvant or observation), adjusted for treatment arm, categorized lymph node burden (reference: ≥4 nodes), and AJCC stage (reference: IIIA–B). A single positive node was associated with significantly reduced recurrence risk (HR = 0.34, 95% CI: 0.13–0.88).

CI, confidence interval; HR, hazard ratio. (后续multivariable analysis可做森林图)


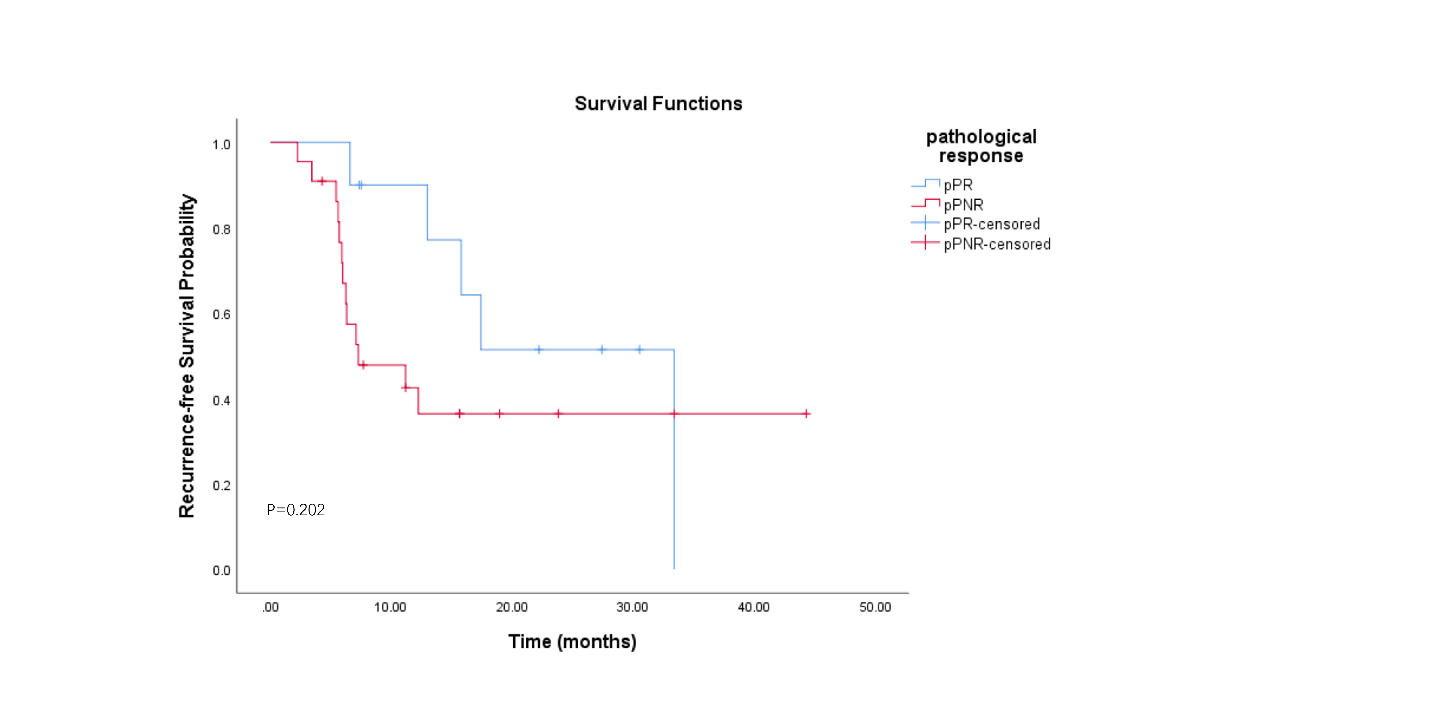


**Supplementary Figure 3** Recurrence-free survival by pathological response to neoadjuvant therapy

Pathological Response (pCR + pPR): 10 cases (31.2%). Pathological No Response (pNR): 22 cases (68.8%). The median RFS could not be reliably estimated for pPR patients as more than 50% remained recurrence-free at the longest follow-up timepoint. However, the mean RFS for pPR patients was 33.37 months. In contrast, pNR patients had a significantly shorter mRFS of 7.27 months (95% CI: 0.28–14.26).

CI, confidence interval; HR, hazard ratio; pNR, pathological non-response; pPR, pathological partial response.


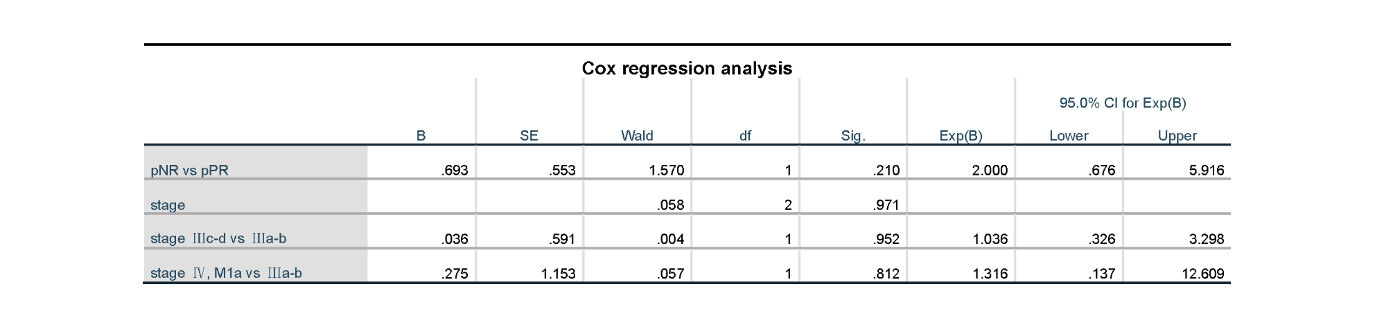


**Supplementary Figure 4** multivariable Cox regression analysis for pathological response

The multivariable analysis assessing the impact of pathological response recurrence risk. Pathological non-response (pNR) was associated with a non-significant increase in hazard compared to partial response (pPR) (HR = 2.00, 95% CI: 0.68–5.92, P = 0.210). Advanced disease stage (IIIc–d or IV M1a) was not significantly associated with increased risk relative to stage IIIa–b.

CI, confidence interval; HR, hazard ratio; pNR, pathological non-response; pPR, pathological partial response.
